# Skinfold thickness affects the isometric knee extension torque evoked by Neuromuscular Electrical Stimulation

**DOI:** 10.1590/bjpt-rbf.2014.0114

**Published:** 2015-09-01

**Authors:** Flávia V. A. Medeiros, Amilton Vieira, Rodrigo L. Carregaro, Martim Bottaro, Nicola A. Maffiuletti, João L. Q. Durigan

**Affiliations:** 1 Faculdade de Educação Física, Universidade de Brasília (UnB), Brasília, DF, Brazil; 2 Faculdade de Ciências da Saúde, Universidade de Brasília (UnB), Brasília, DF, Brazil; 3 Departamento de Fisioterapia, Universidade de Brasília (UnB), Brasília, DF, Brazil; 4 Neuromuscular Research Laboratory, Schulthess Klinik, Zurich, Switzerland

**Keywords:** physical therapy, physical agents, electrotherapy

## Abstract

**BACKGROUND:**

Subcutaneous adipose tissue may influence the transmission of electrical stimuli through to the skin, thus affecting both evoked torque and comfort perception associated with neuromuscular electrical stimulation (NMES). This could seriously affect the effectiveness of NMES for either rehabilitation or sports purposes.

**OBJECTIVE:**

To investigate the effects of skinfold thickness (SFT) on maximal NMES current intensity, NMES-evoked torque, and NMES-induced discomfort.

**METHOD:**

First, we compared NMES current intensity, NMES-induced discomfort, and NMES-evoked torque between two subgroups of subjects with thicker (n=10; 20.7 mm) vs. thinner (n=10; 29.4 mm) SFT. Second, we correlated SFT to NMES current intensity, NMES-induced discomfort, and NMES-evoked knee extension torque in 20 healthy women. The NMES-evoked torque was normalized to the maximal voluntary contraction (MVC) torque. The discomfort induced by NMES was assessed with a visual analog scale (VAS).

**RESULTS:**

NMES-evoked torque was 27.5% lower in subjects with thicker SFT (*p*=0.01) while maximal current intensity was 24.2% lower in subjects with thinner SFT (*p*=0.01). A positive correlation was found between current intensity and SFT (*r*=0.540, *p*=0.017). A negative correlation was found between NMES-evoked torque and SFT (*r*=-0.563, *p*=0.012). No significant correlation was observed between discomfort scores and SFT (r_s_=0.15, *p*=0.53).

**CONCLUSION:**

These results suggest that the amount of subcutaneous adipose tissue (as reflected by skinfold thickness) affected NMES current intensity and NMES-evoked torque, but had no effect on discomfort perception. Our findings may help physical therapists to better understand the impact of SFT on NMES and to design more rational stimulation strategies.

## Introduction

Neuromuscular electrical stimulation (NMES) is widely used to prevent skeletal muscle atrophy and to preserve or improve maximal voluntary strength^1^^-^ 4. The main determinant of NMES effectiveness is the level of torque evoked by NMES^5^^-^ 7. In fact, training-induced gains in muscle strength are directly related to the degree of tension of the muscle contraction elicited by NMES. The degree of tension is strongly influenced by muscle recruitment, which is in turn determined by the intensity of the applied current and by the discomfort associated with the stimulation^5^^,^ 8. Theoretically, NMES current intensity and NMES-induced discomfort should be, respectively, as high and as low as possible^8^^,^ 9, in order to induce the highest levels of muscular tension and consequently to generate the highest evoked torques.

Among the various biological tissues (skin, muscle, and fat), adipose tissue seems to be the more resistant to electrical current^10^. Theoretically, it is necessary to inject high doses to achieve a specific therapeutic range to evoke strong muscle contractions. This is particularly true for individuals with considerable amounts of subcutaneous adipose tissue^10^^,^ 11, such as women^12^. However, this could inevitably lead to discomfort and intolerance to NMES therapy. Unfortunately, few data exist regarding the interference of fat tissue on NMES characteristics under carefully-controlled conditions. For example, although evidence has pointed to the existence of sex-related differences in electrical current thresholds and pain modulation, men and women are often considered together^12^^,^ 13. Moreover, previous studies did not adequately control for menstrual cycle and contraceptive use in female participants, which are known to affect NMES tolerance^14^^-^ 16.

The purpose of this methodological study was to investigate the impact of skinfold thickness (SFT) - as a surrogate of the amount of subcutaneous adipose tissue - on NMES current intensity, NMES-evoked torque, and NMES-induced discomfort in a group of healthy women. First, we categorized subjects according to SFT and hypothesized that those with thicker SFT would require higher NMES current intensity, report higher discomfort levels, and produce lower evoked torque than subjects with thinner SFT. Second, we hypothesized that SFT would be: 1) positively correlated to NMES current intensity; 2) positively correlated to NMES-induced discomfort; and 3) negatively correlated to NMES-evoked torque.

## Method

### Subjects

Sample size was determined a priori using G*Power (version 3.1.3; University of Trier, Trier, Germany) with the level of significance set at *p*=0.05 and power (1-β) = 0.95. We conducted a pilot study with 5 participants to evaluate the effect size for the evoked torque (d=2.62) and current intensity (d=2.09). Based on these a priori calculations, we set the final sample size at n=16, and recruited 20 volunteers considering a 20% drop-out rate. Twenty healthy women (mean±SD age: 22±3 yrs, weight: 59±9 kg, height: 166±7 cm) volunteered to participate in this methodological study. They were categorized in two groups, i.e. thicker SFT (n=10; 20.7 mm) and thinner STF (n=10; 29.4 mm), using the categorization procedure of SPSS (SPSS Inc., Chicago, IL, USA)^11^. Subjects were recruited from Universidade de Brasília (UnB), Brasília, DF, Brazil. The main inclusion criteria were: age between 18 and 35 years; no previous record of muscular disease or traumatic lesions in the knee or any constraints that could interfere with the tests; no current use of analgesics, tranquilizers, antidepressants, or other centrally acting agents; use of oral contraceptives (OC) in the last 3 months; and moderately active (category 2 according to the International Physical Activity Questionnaire). For all the subjects, OC pills contained ethinyl estradiol and gestagen. Subjects who did not feel comfortable with NMES and did not reach the evoked torque level of 30% maximum voluntary contraction (MVC)^5^^,^ 11 were excluded from the study.

Participants were instructed not to take any nutritional supplement or ergogenic aid during the study period. They were also instructed not to perform any form of vigorous or unusual physical activity the day before and after the test. All tests were performed at the same time of the day for each subject. Before participation, each volunteer read and signed a detailed informed consent form approved by the Human Research Ethics Committee (UnB registration 58/13).

### Experimental procedure

Subjects were asked to attend the laboratory on two separate days with a minimum interval of 5 days between visits. The first visit (active phase of the menstrual cycle: day 14-20 of the cycle) served to familiarize subjects with NMES, measure SFT (see "Assessment of SFT" below), and determine the maximal NMES current intensity. The other variables were tested only in the inactive phase of the menstrual cycle. To determine the maximum NMES current intensity, current intensity was gradually increased until the tolerance limit was reached. This current level was reproduced in the second visit. The second visit was in the inactive phase of the menstrual cycle (day 1-7 of the cycle) and was devoted to the assessment of MVC torque, NMES-induced discomfort, and NMES-evoked torque. The maximal NMES current intensity recorded during the first visit was rechecked and eventually further increased. The study was conducted in double-blinded conditions, in which both the volunteer and the NMES operator did not know which current was applied.

### Assessment of SFT

SFT was measured to the nearest 0.5 mm with a Lange skinfold caliper (Cambridge Scientific Industries, Cambridge, MD, USA). SFT was measured on the anterior aspect of the dominant thigh at 50% of the distance between the inguinal crease and the upper border of the patella^17^. The average SFT of three consecutive measurements was retained.

### Assessment of MVC and NMES-evoked torque

All the procedures were performed on the dominant leg (leg used to kick a ball). Subjects were positioned into the chair of an isokinetic dynamometer (System 3, Biodex Medical Systems, Shirley, NY, USA) with the hip at 90° and the knee joint at 60° of flexion. The axis of the dynamometer was aligned with the axis of rotation of the knee. The lever arm of the dynamometer was parallel to the anterior aspect of the tibia, with the lower edge of the pad positioned ~3 cm proximal to the lateral malleolus. The trunk, waist, and thigh were stabilized using straps. Calibration of the dynamometer was performed before each testing session according to the manufacturer's specifications.

Before starting the test, the subjects had the thigh shaved and the skin cleansed with isopropyl alcohol. Subsequently, they completed a warm-up consisting of several submaximal concentric knee extensions at an angular velocity of 180°/s. Then, isometric MVC torque was assessed as follows: subjects were requested to perform three 10-s MVC separated by rest periods of 3 min. They received visual feedback in real time and verbal encouragements to perform a maximal effort with a progressive force build-up. Only the highest MVC torque was retained. Finally the isometric knee extension torque evoked by NMES was measured, and it was consistently normalized to the MVC torque.

The stimulator (Neurodyn 2.0, Ibramed, Amparo, SP, Brazil) was connected to isolated cables, and the cables were connected to two pairs of self-adhesive electrodes each measuring 50x50 mm (ValuTrode, Axelgaard, Fallbrook, CA, USA). For channel 1, the distal electrode was placed at a point 80% the distance between the anterior superior iliac spine and the medial condyle of the femur; the proximal electrode was placed 10-15 cm above the distal electrode on the vastus medialis muscle^18^. For channel 2, the distal electrode was positioned at a point 2/3 the distance between the anterior superior iliac spine and the lateral border of the patella on the vastus lateralis muscle and the proximal electrode was placed 10-15 cm above the distal electrode on the vastus lateralis muscle^18^.

We used pulsed current (biphasic symmetric) with a frequency of 50 Hz and pulse duration of 500 μs. Current intensity was progressively increased from 0 mA at a rate of about 1 mA/s up to the maximal tolerable intensity. Three NMES-induced contractions of 10 s were completed to minimize fatigue. Current was delivered with a 3-s ramp up, a decay of 1 s, and a rest interval of 3 s between contractions. Only the highest torque induced by electrical stimulation was retained. Subjects were instructed to relax fully during NMES so that the evoked torque could be measured with minimal or no voluntary contribution. All physical parameters of the stimulator were checked using a digital oscilloscope (DS1052E, Rigol Technologies, Beaverton, OR, USA).

### Assessment of NMES-induced discomfort

The maximal discomfort level was assessed by a 10-cm visual analog scale (VAS) with 0 representing "no discomfort" and 10 representing "maximum tolerated discomfort". The VAS was presented to the participants immediately after the assessment of NMES-evoked torque (after the third evoked contraction) and they were asked to point a mark on a VAS to rate the level of discomfort.

### Statistical analysis

Normality was consistently checked using the Shapiro-Wilk test. Two-tailed independent *t-*tests were used to examine differences in SFT, NMES current intensity, and NMES-evoked torque between subjects with thicker vs. thinner SFT, while the Mann-Whitney test was used for NMES-induced discomfort. Two-tailed Pearson's product-moment correlation coefficients (r) were calculated to determine the strength of the association between (1) SFT and NMES current intensity and between (2) SFT and NMES-evoked torque. The non-parametric two-tailed Spearman's correlation coefficient (r_s_) was calculated to evaluate the association between SFT and NMES-induced discomfort. Correlation coefficients between ±0.1 and ±0.3, ±0.4 and ±0.6, and >0.7 were considered weak, moderate, and strong, respectively. All statistical analyses were performed using SPSS (SPSS Inc., Chicago, IL, USA). Significance was set at *p<*0.05 for all the procedures.

## Results

**Figure 1.**
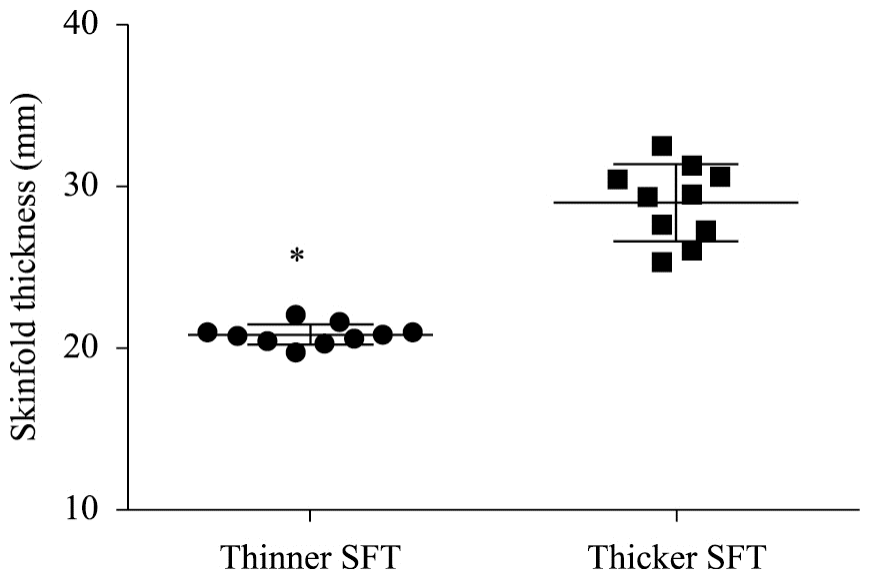
Mid-thigh skinfold thickness by subgroup (subjects with thinner vs. thicker skinfold thickness). Single data points are presented, with mean and SD as the error bars. The difference in SFT between the two groups was significant (* p=0.001).

The mean±SD MVC torque was 178.4±9.5 Nm. The comparison between the two groups (thicker vs. thinner SFT) is presented in Figure 1 and Table 1. Both SFT and maximal NMES current intensity were significantly higher in subjects with thicker SFT than in those with thinner SFT (*p<*0.0001 and *p*=0.03, respectively), with a mean inter-group difference of 29% and 24%, respectively. The maximal NMES-evoked torque was 28% lower in subjects with thicker SFT than in those with thinner SFT (*p<*0.01). The maximal level of discomfort induced by NMES did not differ significantly between two groups (*p=*0.15).

**Table 1.** Maximal NMES current intensity, NMES-evoked torque, and NMES-induced discomfort by SFT level.

	Thinner SFT	Thicker SFT	*P* value
NMES current intensity (mA)	73.8 (±4.6)	97.3 (±4.8)^*^	0.03
NMES-evoked torque (% MVC)	73.4 (±5.9)^*^	53.2 (±5.2)	0.01
NMES-induced discomfort (0-10)	6.1 (±2.6)	7.2 (±3.3)	0.8

NMES: neuromuscular electrical stimulation; SFT: skinfold thickness; MVC: maximal voluntary contraction. *p=statistical significance. Data are mean±standard deviation.

**Figure 2.**
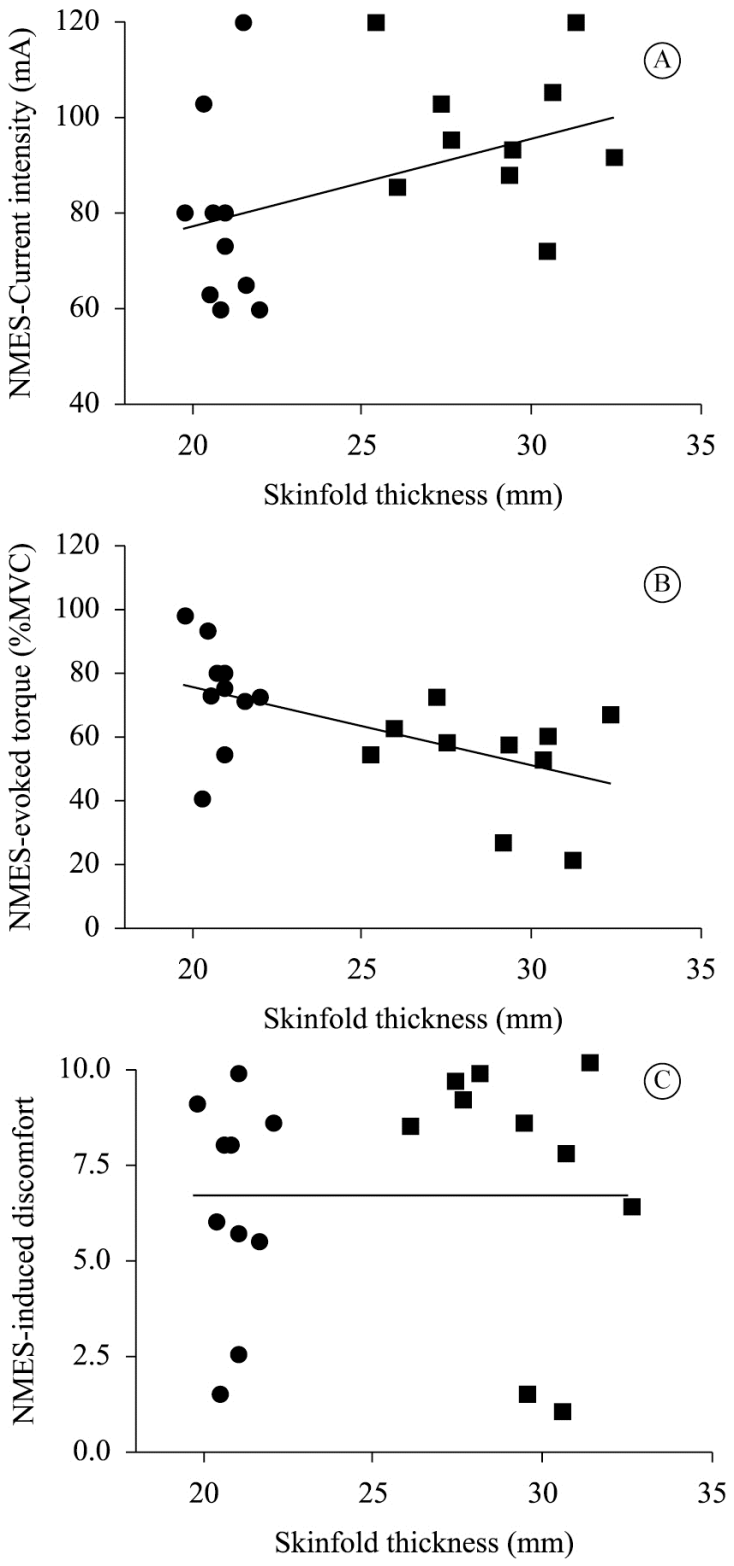
(A) Correlation between NMES current intensity (mA) and mid-thigh skinfold thickness (r=0.540, p=0.017). (B) Correlation between NMES-evoked torque (%MVC) and mid-thigh skinfold thickness (r=-0.563, p=0.012). (C) Correlation between NMES-induced discomfort (VAS 0-10) and mid-thigh skinfold thickness (rs=0.15, p=0.53).

SFT showed a moderate positive correlation with NMES current intensity (*r*=0.540, *p*=0.017; Figure 2A) and a moderate negative correlation with NMES-evoked torque (*r*=-0.563, *p*=0.012; Figure 2B). No significant correlation was observed between SFT and NMES-induced discomfort (r=0.15, *p*=0.53; Figure 2C).

## Discussion

The main findings of this methodological study support the assumption that the amount of subcutaneous adipose tissue, as estimated by means of SFT assessment, interferes with the current intensity necessary to optimize the effectiveness of NMES, but does not affect the level of perceived discomfort. In fact, our main results were that women with thicker SFT required higher NMES current intensities but generated lower evoked torques (~53% MVC) compared to women with thinner SFT (~73% MVC). Considering that the level of torque evoked by NMES is the main determinant of NMES effectiveness^6^^,^ 19, it is reasonable to expect higher NMES training-induced strength gains in individuals with small amounts of subcutaneous fat. The present results may help physical therapists to design more rational stimulation paradigms in an attempt to optimize the clinical application of NMES therapy.

We clearly demonstrated a dependency of NMES current intensity and NMES-evoked torque on SFT. In line with our results, Miller et al.^11^showed that, to produce a similar level of evoked torque, subjects with thicker SFT required stronger NMES currents than subjects with thinner SFT. They also observed a positive correlation between NMES current intensity and SFT^11^. Furthermore, Tomazin et al.^20^ confirmed that progressively larger adipose thicknesses at the site of femoral nerve were associated with progressively lower peak twitch forces induced by magnetic stimulation. They suggested that larger adipose thickness reduced current diffusion in a dose-response manner, probably due to the increased distance from the stimulating coil to the femoral nerve cell membrane^20^. In fact, subcutaneous adipose tissue has low electrical conductivity^21^, thus limiting the spread of current flow and reducing skin current diffusion towards the muscle^22^, which could in turn affect muscle activation. The thicker the fat layer is, the greater the resistance and the longer the distance between the stimulating electrode and the motor unit branches. This largely explains why individuals with thicker SFT at the mid-thigh level required stronger currents for triggering quadriceps muscle contractions^11^^,^ 20.

Although additional factors may influence NMES use in subjects with thicker SFT^23^, this study suggests that stimulation efficacy should be consistently verified before any application of NMES, particularly in women and in overweight subjects. A possible solution would be to use very large electrodes or to avoid muscle areas with thick SFT (e.g. rectus femoris for NMES of the quadriceps). Then, the efficacy of NMES could be preserved while reducing the current density at skin level, thereby minimizing the associated subjective discomfort^24^^,^ 25. In addition, physical therapists should systematically ascertain that the NMES device they use is able to generate enough current to achieve an adequate NMES therapeutic target (i.e. evoked torque between 25 and 50% MVC).

In relation to the subjective sensations elicited by NMES, our findings showed no association between the amount of SFT and discomfort perception. This suggests that subcutaneous adipose tissue does not seem to interfere with the perception of NMES-induced discomfort. Interestingly, this sensation is not exclusively related to physical stimulation characteristics and nociceptor activation, but also involves psychological and social aspects^23^^,^ 26. There is some evidence to suggest that the degree of discomfort elicited by NMES is related to affective and emotional past experiences. Therefore, subjects who have a negative experience with electrical stimuli, with regard to fearful sensations or heightened sense of anxiety, may have a lower tolerance to the current^23^. This suggests that, regardless of SFT, the discomfort elicited by NMES is subject to large inter-individual differences^12^^,^ 26 that are difficult to control.

It is important to emphasize that, in the present study, we examined the impact of SFT on NMES current intensity, NMES-evoked torque, and NMES-induced discomfort in women using OC^14^. The experimental session was conducted in the inactive phase of the menstrual cycle (day 1-7 of the cycle), since testing at different phases of the cycle may have influenced the discomfort elicited by NMES^14^^,^ 15 ^,^ 27. Previous studies did not account for potential sex-related confounders, i.e. subjects from both genders were considered as a unique sample, and the phase of the menstrual cycle was not adequately controlled. Future investigations are needed to compare NMES-evoked torque and discomfort between the different phases of the menstrual cycle in both OC users and non-users.

One limitation of the present study is that we only focused on the use of NMES in young and healthy women; therefore, additional comparative studies are required to examine the impact of SFT in elderly individuals and in clinical populations of both genders. Studies with different patient groups are required to understand how physical and muscle dysfunction may affect discomfort and NMES-evoked torque in relation with SFT. Another limitation is that we measured SFT with a low-cost though relatively straightforward and valid technique^28^^,^ 29, which is not necessarily the most accurate methodology for estimating the amount of subcutaneous fat (as opposed to ultrasonography and segmental bioelectrical impedance analysis that are currently quite accessible). Furthermore, potential variations in impedance, skin temperature, and muscle thickness that could have occurred between and within the testing sessions^22^^,^ 30 were not controlled.

In conclusion, the amount of subcutaneous adipose tissue affected both NMES current intensity and NMES-evoked torque of the knee extensor muscles in healthy women, but did not influence the level of perceived discomfort. These results suggest that subcutaneous fat is an important variable that should receive more attention for an optimal application of NMES therapy in clinical settings.
